# A new ^68^Ga-labeled somatostatin analog containing two iodo-amino acids for dual somatostatin receptor subtype 2 and 5 targeting

**DOI:** 10.1186/s13550-020-00677-3

**Published:** 2020-08-05

**Authors:** Rosalba Mansi, Karim Abid, Guillaume P. Nicolas, Luigi Del Pozzo, Eric Grouzmann, Melpomeni Fani

**Affiliations:** 1 Division of Radiopharmaceutical Chemistry, Clinic of Radiology and Nuclear Medicine, University Hospital Basel, Petersgraben 4, 4031 Basel, Switzerland; 2 Catecholamine and Peptides Laboratory, Department of Laboratories, University Hospital of Lausanne, Lausanne, Switzerland; 3 Division of Nuclear Medicine, Clinic of Radiology and Nuclear Medicine, University Hospital Basel, Basel, Switzerland

**Keywords:** Somatostatin receptor subtypes, Somatostatin agonists, SST2, SST5, ^68^Ga, PET, Neuroendocrine tumors, Iodo-amino acids

## Abstract

**Background:**

Somatostatin receptor (SST) targeting, specifically of the subtype 2 (SST2), with radiolabeled somatostatin analogs, is established for imaging and treatment of neuroendocrine tumors. Owing to the concomitant and heterogeneous expression of several subtypes on the same tumor, analogs targeting more subtypes than SST2 potentially target a broader spectrum of tumors and/or increase the uptake of a given tumor. The analog ST8950 ((4-amino-3-iodo)-d-Phe-c[Cys-(3-iodo)-Tyr-d-Trp-Lys-Val-Cys]-Thr-NH_2_), bearing 2 iodo-amino acids, exhibits sub-nanomolar affinity to SST2 and SST5. We report herein the development and preclinical evaluation of DOTA-ST8950 labeled with ^68^Ga, for imaging SST2- and SST5-expressing tumors. Comparative in vitro and in vivo studies were performed with the de-iodinated DOTA-ST8951 ((4-amino)-d-Phe-c[Cys-Tyr-d-Trp-Lys-Val-Cys]-Thr-NH_2_) and with the reference compounds DOTA-TATE (SST2 selective) and DOTA-NOC (for SST2 and SST5).

**Results:**

Compared with ^nat^Ga-DOTA-NOC, ^nat^Ga-DOTA-ST8950 exhibited higher affinity to SST2 and SST5 (IC_50_ (95%CI), nM = 0.32 (0.20–0.50) and 1.9 (1.1–3.1) vs 0.70 (0.50-0.96) and 3.4 (1.8-6.2), respectively), while ^nat^Ga-DOTA-ST8951 lost affinity for both subtypes. ^nat^Ga-DOTA-ST8950 had the same potency for inducing SST2-mediated cAMP accumulation as ^nat^Ga-DOTA-TATE and slightly better than ^nat^Ga-DOTA-NOC (EC_50_, nM = 0.46 (0.23–0.92) vs 0.47 (0.15–1.5) vs 0.59 (0.18–1.9), respectively). [^67^Ga]Ga-DOTA-ST8950 had a similar internalization rate as [^67^Ga]Ga-DOTA-NOC in SST2-expressing cells (12.4 ± 1.6% vs 16.6 ± 2.2%, at 4 h, *p* = 0.0586). In vivo, [^68^Ga]Ga-DOTA-ST8950 showed high and specific accumulation in SST2- and SST5-expressing tumors, comparable with [^68^Ga]Ga-DOTA-NOC (26 ± 8 vs 30 ± 8 %IA/g, *p* = 0.4630 for SST2 and 15 ± 6 vs 12 ± 5 %IA/g, *p* = 0.3282, for SST5, 1 h p.i.) and accumulation in the SST-positive tissues, the kidneys and the liver. PET/CT images of [^68^Ga]Ga-DOTA-ST8950, performed in a dual HEK-SST2 and HEK-SST5 tumor xenografted model, clearly visualized both tumors and illustrated high tumor-to-background contrast.

**Conclusions:**

[^68^Ga]Ga-DOTA-ST8950 reveals its potential for PET imaging SST2- and SST5-expressing tumors. It compares favorably with the clinically used [^68^Ga]Ga-DOTA-NOC in terms of tumor uptake; however, its uptake in the liver remains a challenge for clinical translation. In addition, this study reveals the essential role of the iodo-substitutions in positions 1 and 3 of [^68^Ga]Ga-DOTA-ST8950 for maintaining affinity to SST2 and SST5, as the de-iodinated [^68^Ga]Ga-DOTA-ST8951 lost affinity for both receptor subtypes.

## Introduction

Nuclear imaging of somatostatin receptor (SST)-expressing tumors is established for the detection of neuroendocrine tumors (NETs) and their metastases. SST scintigraphy, using [^111^In][In-diethylenetriaminepentaacetic acid^0^]-octreotide ([^111^In]In-DTPA^0^-octreotide, Octreoscan®), has covered this medical need since the 1990s. Nowadays, positron emission tomography (PET) with improved octreotide-based analogs labeled with ^68^Ga represents the state of the art. The most widely used analogs consist of the [^68^Ga][Ga-1,4,7,10-tetraazacyclododecane-1,4,7,10-tetraacetic acid^0^,Tyr^3^]-octreotide ([^68^Ga]Ga-DOTA-TOC, SOMAKIT TOC®) with a high affinity for SST2 and a weaker affinity for SST5 (IC_50_ = 2.5 ± 0.5 and 73 ± 21 nM, respectively) and the high affinity SST2-selective [^68^Ga][Ga-DOTA^0^,Tyr^3^,Thr^8^]-octreotate ([^68^Ga]Ga-DOTA-TATE, NETSPOT®) with an IC_50_ of 0.20 ± 0.04 nM [1].

Although the majority of NETs expresses SST2, a low and heterogeneous expression has been reported in approximately 20–30% of cases [2–5]. This is associated with an inherent worse disease prognosis, a lower sensitivity in imaging and an ineffective therapy with SST2-specific analogs due to inadequate tumor targeting [6]. Among the five SST subtypes (SST1-SST5), SST5 is concomitantly expressed at high density in 70–100% of gastroenteropancreatic neuroendocrine tumors (GEP-NETs), breast cancer and in growth hormone (GH)-secreting pituitary adenomas [7–9].

The only clinically used analog for imaging of different SST subtypes is the octreotide-based [^68^Ga][Ga-DOTA^0^,1-Nal^3^]-octreotide ([^68^Ga]Ga-DOTA-NOC), with high affinity for SST2 and SST5 and lower affinity for SST3 [10–12]. The cyclohexapeptide pasireotide (Signifor®, formerly known as SOM230) [13, 14] is another analog with an affinity for SST2, SST3, and SST5 that have been evaluated preclinically with ^68^Ga ([^68^Ga]Ga-DOTA-SOM230 [15, 16] or [^68^Ga]Ga-DOTA-PA1 [17]). Other preclinical attempts for combined targeting of different subtypes involve ^111^In-labeled analogs of (a) NOC [18], (b) carbocyclic octapeptides based on the cyclic KE108 with a non-disulfide 8 member ring [19]; (c) 14mer and *pseudo*-14mer cyclic somatostatin-14 (SS-14) mimics, with ring-size of 12, 9, 8, and 6 amino acids [20, 21], and (d) somatostatin-28 (SS-28) modified at positions 8, 22, and 25 [22]. All the abovementioned radiotracers showed certain limitations, with [^68^Ga]Ga-DOTA-NOC being, so far, the only one used in the clinic.

**Fig. 1:**
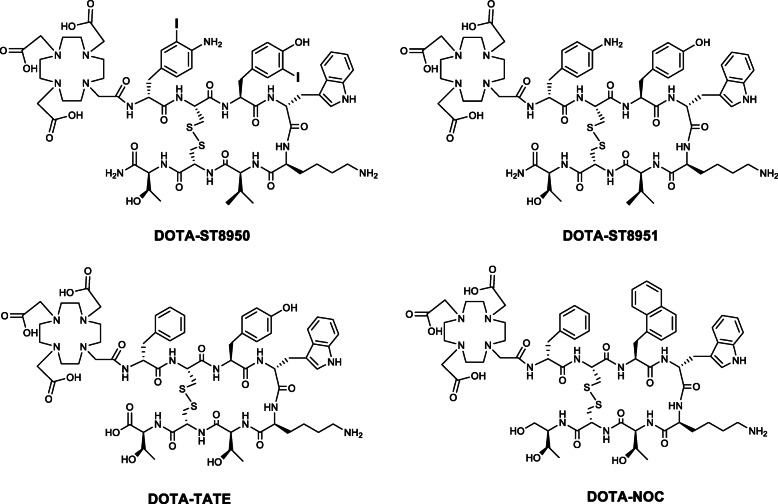
The structural formulae of the four DOTA-conjugated somatostatin analogs (DOTA-ST8950: DOTA-(4-amino-3-iodo)-d-Phe-c[Cys-(3-iodo)-Tyr-d-Trp-Lys-Val-Cys]-Thr-NH_2_, DOTA-ST8951: DOTA-(4-amino)-d-Phe-c[Cys-Tyr-d-Trp-Lys-Val-Cys]-Thr-NH_2_, DOTA-NOC: DOTA-d-Phe-c[Cys-1-NaI-d-Trp-Lys-Thr-Cys]Thr(ol) and DOTA-TATE: DOTA-d-Phe-c[Cys-Tyr-d-Trp-Lys-Thr-Cys]Thr)

We are interested in developing somatostatin analogs with high affinity to SST2 and SST5 for targeting a broader spectrum of tumors and/or increasing the tumor uptake, when both receptor subtypes are concomitantly present. A library of disulfide-bridged octapeptides based on RC-121 (d-Phe-c(Cys-Tyr-d-Trp-Lys-Val-Cys)-Thr-NH_2_) [23] that contains synthetic amino acids and modifications at positions 1, 3, and 8 was developed by Moore et al [24]. Out of this library, ST8950 (Fig. 1, peptide #9 in reference [24]) bearing the 2 iodo-amino acids 4-amino-3-iodo-phenylalanin in position 1 and 3-iodo-tyrosine in position 3 exhibited sub-nanomolar affinity to both SST2 and SST5 and showed to be as potent as the natural SS-14 in the inhibition of growth hormone and prolactin release. We previously reported that ST8950 (AP102 in references [25, 26]) has an intermediate agonistic potency between octreotide and pasireotide at SST2 and SST5 level [25] and reduces growth hormone secretion without causing hyperglycemia (a known undesirable effect of pasireotide) in a healthy rat model [26]. We aimed to develop and evaluate ^68^Ga-labeled ST8950 for PET imaging of SST2- and SST5-expressing tumors and we used DOTA as a chelator. Knowing that modifications such as chelator conjugation and (radio)metallation impact on affinity and whole-body distribution of radiolabeled somatostatin analogs [10, 27], with sometimes unexpected outcome, we decided to include in our study a second analog as an alternative. We chose the de-iodinated ST8951 (Fig. 1, peptide #2 in reference [24]) that exhibits also good affinity to SST2 and SST5 in an attempt to assess, additionally, the influence of the iodo-substitution on the Ga-DOTA conjugates.

## Methods

### (Radio)metallated peptide conjugates

DOTA-ST8950 and DOTA-ST8951 were custom-made by PolyPeptide (San Diego, USA). The reference conjugates DOTA-TATE and DOTA-NOC were synthesized following Fmoc-solid-phase peptide synthesis, purified by preparative reverse phase high-performance liquid chromatography (RP-HPLC) and characterized by electrospray ionization mass spectroscopy (ESI-MS). The structural formulae of all four conjugates are presented in Fig. 1.

The ^nat^Ga complexes of the four conjugates were prepared using 2.5-fold excess of ^nat^Ga(NO_3_)_3_ × 9H_2_O in ammonium acetate buffer, 0.2 M, pH 4 at 95 °C for 30 min. Free metal ions were eliminated via SepPak C-18 cartridge (Waters), pre-conditioned with methanol and water. The reaction mixture was loaded and the free ^nat^Ga was eluted with water while the metallo-peptides were eluted with ethanol, evaporated to dryness, re-dissolved in water and lyophilized.

^67^Ga-labeled conjugates were prepared by reacting 6 nmol of the corresponding conjugate in 250 μL Na-acetate buffer (0.2 M, pH 4.1) with [^67^Ga]GaCl_3_ (30-50 MBq, Mallinckrodt) at 95 °C for 30 min. DOTA-ST8950 and DOTA-NOC were labeled with ^68^Ga in an automatic Modular-Lab Pharm Tracer module (Eckert & Ziegler). Briefly, the ^68^Ge/^68^Ga-generator (IGG100, Eckert & Ziegler) was eluted with 7 mL HCl 0.1 N and the eluate (~ 800 MBq) was loaded onto a cation exchange column (Strata-XC, Phenomenex). ^68^Ga^3+^ was eluted with 800 μL of acetone/HCl (97.6%/0.02 N) in a vial containing 2 mL Na-acetate buffer (0.2 M, pH 4.0) and 10 nmol of the conjugate. The stability of [^68^Ga]Ga-DOTA-ST8950 was evaluated for 1 h after production at room temperature (RT), without any formulation of the product.

The quality control and the stability study were performed by analytical RP-HPLC on Phenomenex Jupiter Proteo 90 Å C12 (250 × 4.6 mm) column (eluents: *A* = H_2_O (0.1%TFA), *B* = acetonitrile (0.1% TFA); gradient: 95–50% A in 15 min; flow rate: 1.5 mL/min). ESI-MS was carried out with ESI Bruker Esquire 3000 plus (Bruker Daltonics).

### Cell lines, affinity studies, and functional assays

Human Embryonic Kidney (HEK293) cells (a kind gift from Dr. A. Mühlethaler-Mottet, Pediatric Hematology-Oncology Unit, Lausanne University Hospital, Switzerland) were stably transfected with plasmids encoding the human SST2 and SST5 (HEK-SST2 and HEK-SST5) and cultivated as previously described [25]. Non-transfected HEK cells were used as a negative control.

The binding affinities of ^nat^Ga-DOTA-ST8950 and ^nat^Ga-DOTA-ST8951, in comparison to ^nat^Ga-DOTA-TATE and ^nat^Ga-DOTA-NOC, were determined on HEK-SST2 and HEK-SST5 cells. SS-14, octreotide, and pasireotide were used as reference compounds. ^125^I-labeled SS-14 (81.4 TBq/mmol, Perkin Elmer) was used as a radioligand for the competition binding assays. Binding assays were performed as described previously [25].

cAMP accumulation experiments on HEK-SST2 and HEK-SST5 cells were performed with cAMP direct immunoassay kit (colorimetric, K371, BioVision) as described previously [25].

### Log *D* measurement

Log *D* (pH = 7.4) was determined by the “shake-flask” method. To a pre-saturated mixture of 500 μL n-octanol and 500 μL of phosphate-buffered saline (PBS) at pH 7.4, 10 μL of 1 μM of ^67^Ga-labeled conjugates was added. The solutions were vortexed for 1 h to reach equilibrium and then centrifuged (3000 rpm) for 10 min. From each phase, 100 μL was removed and measured in a γ-counter. The partition coefficient was calculated as the average of the logarithmic values (*n* = 3) of the ratio between the radioactivity in the organic and the PBS phase.

### In vitro characterization

For cell experiments, stably SST2- and SST5-expressing cells were seeded in 6-well plates (10^6^ cells/well) and incubated overnight with Dulbecco’s modified Eagle’s medium (DMEM) with 1% fetal bovine serum (FBS, Biochrom GmbH, Merck Millipore) to obtain a good cell adherence. The plates were pre-treated with a solution of 10% poly-lysine to promote cell adherence.

### Internalization assays

The cells were washed with PBS and incubated with fresh medium (DMEM with 1% FBS) for 1 h at 37 °C/5% CO_2_. [^67^Ga]Ga-DOTA-ST8950, [^67^Ga]Ga-DOTA-ST8951, [^67^Ga]Ga-DOTA-TATE, or [^67^Ga]Ga-DOTA-NOC (2.5 nM) were added to the medium, and the cells were incubated for 0.5, 1, 2, and 4 h at 37 °C/5% CO_2_ (in triplicates). The internalization process was stopped by removing the medium and washing the cells with ice-cold PBS, followed by 2 × 5 min treatment with ice-cold glycine solution (0.05 M, pH 2.8), to distinguish between cell surface-bound (acid releasable) and internalized (acid resistant) radio-conjugate. Finally, the cells were detached with 1 M NaOH at 37 °C. To determine non-specific uptake, selected wells were incubated with the radio-conjugate in the presence of 1000-fold excess of SS-14. Internalization and bound rate are expressed as a percentage of the applied radioactivity.

### Off-rate experiments

HEK-SST2 cells were incubated with [^67^Ga]Ga-DOTA-ST8950, [^67^Ga]Ga-DOTA-TATE, or [^67^Ga]Ga-DOTA-NOC (2.5 nM) for 2 h. The medium was removed and the wells were washed with ice-cold PBS. The surface-bound radio-conjugate was removed with a glycine solution (pH 2.8) on ice, as described above. Cells were then incubated again at 37 °C with a fresh medium. At 10, 20, 30, 60, 120, and 240 min, the medium was removed for quantification of radioactivity and replaced with a fresh 37 °C medium. At the end of the experiment, the cells were detached with 1 M NaOH and collected for quantification of the radioactivity.

### In vivo evaluation

The Veterinary Office (Department of Health) of the Cantonal Basel-Stadt approved the animal experiments (approval no. 2799) in accordance with the Swiss regulations for animal treatment. Female athymic Nude-*Foxn1*^*nu*^*/Foxn1*^*+*^ mice (Envigo, The Netherlands), 4–6 weeks old, were inoculated subcutaneously with 10^7^ HEK-SST2 cells on the right shoulder and 10^7^ HEK-SST5 cells on the left shoulder, suspended in 100 μL sterile PBS. The tumors were allowed to grow for 2–3 weeks until reach an average volume of 100 mm^3^, considering both tumor types. The average tumor mass was 0.22 g (0.14–0.31 g) for SST2 tumors and 0.13 g (range 0.08–0.21) for SST5 tumors. For the biodistribution (cohorts of *n* = 3–8 mice) and imaging studies, the mice were euthanized by keeping them in a CO_2_ chamber 2 min, followed by a slow increase of the concentration of CO_2_ gas. The mice with the largest tumors were used for PET imaging.

### Biodistribution studies of [^68^Ga]Ga-DOTA-ST8950 and [^68^Ga]Ga-DOTA-NOC

Quantitative biodistribution studies were conducted with [^68^Ga]Ga-DOTA-ST8950 (100 μL/100 pmol/5 MBq) at 1 and 2 h p.i. Biodistribution of [^68^Ga]Ga-DOTA-NOC was assessed 1 h p.i. for comparison. The organs of interest were collected, rinsed, blotted, weighed, and counted in a γ-counter. The results are expressed as the percentage of injected activity per gram (%IA/g) obtained by extrapolation from counts of an aliquot taken from the injected solution as a standard. The specificity of uptake of [^68^Ga]Ga-DOTA-ST8950 was assessed 1 h p.i in HEK-SST-negative xenografted mice.

### PET/CT imaging of [^68^Ga]Ga-DOTA-ST8950 and [^68^Ga]Ga-DOTA-NOC

[^68^Ga]Ga-DOTA-ST8950 or [^68^Ga]Ga-DOTA-NOC (100 μL/100 pmol/5 MBq) was administered to mice bearing dual HEK-SST2 and HEK-SST5 tumors. One-hour p.i. the mice were euthanized, and the bladder was emptied by gently pressing with hands around the bladder area. The excess urine was soaked by cotton, followed by repetitive cleaning of the area with ethanol. The mice were scanned for 60 min using a human PET/CT scanner (Discovery STE, GE Medical Systems). A scout scan (180°, 10 mA, 120 kV) was performed to establish a protocol for all other scans. CT scans were acquired with a minimum slice thickness of 0.625 mm, pitch 1.375:1) and the highest possible tube current for these settings (320 mA @ 120 keV). PET emission events were collected in 3D scanning mode (septa out) over 60 min. Images were corrected for the decay of ^68^Ga and random events and reconstructed using the manufacturer’s 3D OSEM algorithm to 47 slices (display FOV = 6.4 cm, 128 × 128 matrix, resulting pixel size = 0.5 mm), once for each mouse separately in the center of the reconstruction cylinder. The in vivo images are presented as fused images of PET maximum intensity projection (MIP) and CT.

### Data analysis

Statistical analysis was performed by unpaired two-tailed *t* test using GraphPad Prism 7 software (GraphPad Software Inc.). *P* values of < 0.05 were considered significant.

## Results

### (Radio)metallated peptide conjugates, stability, and lipophilicity

All (metallated) conjugates were used with > 96% purity. The analytical data are reported in Table 1. The radiochemical yield of the ^68^Ga-preparations (non-isolated, estimated by radio-HPLC) was ≥ 98%, with a radiochemical purity ≥ 95% and an apparent molar activity of 50 MBq/nmol. [^68^Ga]Ga-DOTA-ST8950 was stable after 1 h at a room temperature (radiochemical purity remained ≥ 97%).

**Table 1:** Analytical data of the DOTA conjugates and of their corresponding ^nat^Ga-complexes

Compounds	Purity(%)	MW (calculated)	MW (observed)	HPLC (t_**r**_ min)
DOTA-ST8950	100	1699.5	1700.1	10.51
DOTA-ST8951	98	1447.7	1448.2	8.56
DOTA-NOC	96	1454.6	1456.2	11.00
DOTA-TATE	100	1435.6	1436.2	9.05
^nat^Ga-DOTA-ST8950	98	1768.5	1768.0	10.74
^nat^Ga-DOTA-ST8951	97	1516.7	1516.1	8.55
^nat^Ga-DOTA-NOC	97	1523.6	1524.1	11.40
^nat^Ga-DOTA-TATE	98	1504.6	1504.1	9.50

*MW* molecular weight, *t*_*r*_ retention time

[^67^Ga]Ga-DOTA-ST8950 was more lipophilic (log *D* = − 1.0 ± 0.1) than [^67^Ga]Ga-DOTA-NOC (log *D* = − 1.6 ± 0.1), while [^67^Ga]Ga-DOTA-TATE showed the highest hydrophilicity with a log *D* = − 3.0 ± 0.1.

### Affinity studies

The results are summarized in Table 2. Values regarding SS-14, ST8950, octreotide, and pasireotide have been published in our recent study [25], but were measured head-to-head with all ^nat^Ga-metallated compounds presented here. The IC_50_ of the natural SS-14 is in sub-nanomolar level for both receptor subtypes, while the IC_50_ of ST8950 correlates with the results reported by Moore et al. [24]. Conjugation of the chelate ^nat^Ga-DOTA to ST8950 did not alter its binding affinity to SST2 (IC_50_ (95% CI): 0.32 (0.20–0.50) nM for ^nat^Ga-DOTA-ST8950 vs 0.28 (0.19–0.42) nM for ST8950), but reduced by more than a factor of 2 its affinity to SST5 (IC_50_: 1.9 (1.1–3.1) vs 0.77 (0.48–1.2) nM, respectively). In comparison to ^nat^Ga-DOTA-TATE, ^nat^Ga-DOTA-ST8950 exhibited a lower affinity to SST2 (IC_50_: 0.15 (0.11–0.19) vs 0.32 (0.20–0.50) nM, respectively), but ^nat^Ga-DOTA-TATE was unable to bind to SST5. Compared with ^nat^Ga-DOTA-NOC, ^nat^Ga-DOTA-ST8950 exhibited higher affinity for SST2 and SST5 (IC_50_: 0.70 (0.50–0.96) vs 0.32 (0.20–0.50) nM and 3.4 (1.8–6.2) vs 1.9 (1.1–3.1) nM, respectively [25]). Surprisingly, the de-iodinated analog ^nat^Ga-DOTA-ST8951 demonstrated diminished affinities for SST2 and SST5, compared to ^nat^Ga-DOTA-ST8950 and the reference compounds.

**Table 2:** Affinity toward SST2 and SST5 of all four ^nat^Ga-DOTA-conjugates compared to reference somatostatin analogs

Compounds	SST2	SST5
	IC_**50**_, nM (95% CI)	IC_**50**_, nM (95% CI)
Somatostatin-14^* (1)^	0.11 (0.08–0.15)	0.35 (0.22–0.55)
^nat^Ga-DOTA-ST8950	0.32 (0.20–0.50)	1.9 (1.1–3.1)
^nat^Ga-DOTA-ST8951	7.5 (5.2–11)	24 (14–43)
^nat^Ga-DOTA-TATE	0.15 (0.11–0.19)	69 (28–168)
^nat^Ga-DOTA-NOC	0.70 (0.50–0.96)	3.4 (1.8–6.2)
ST8950 ^(1)^	0.28 (0.19–0.42)	0.77 (0.48–1.2)
Octreotide ^(1)**^	0.24 (0.12–0.48)	17 (12–24)
Pasireotide ^(1)**^	3.1 (2.0–4.9)	0.20 (0.11–0.35)

Experiments were performed in 3 to 5 separate sessions in duplicate

^*^Somatostatin-14 is the natural ligand and was used as control

**Octreotide and pasireotide were used for comparison, additionally to ^nat^Ga-DOTA-TATE and ^nat^Ga-DOTA-NOC

^(1)^From [25]

### Functional assays

The results are summarized in Table 3. The EC_50_ values of SS-14, ST8950, octreotide, and pasireotide were published in our recent study [25], while all ^nat^Ga-DOTA-conjugates are presented here. ST8950 was found to be a highly potent agonist of SST2 (EC_50_ (95% CI) = 0.29 (0.12–0.67) nM (similar to natural SS-14) [25]), but with a lower potency toward SST5 (EC_50_ = 8.5 (3.7–19) nM). However, ST8950 exhibited an intermediate agonistic potency on SST5 between pasireotide and octreotide, the latter being almost inactive on SST5. Introduction of the chelate ^nat^Ga-DOTA reduces the agonistic potency for SST2 by a factor of 1.6 (EC_50_ = 0.46 (0.23–0.92) nM for ^nat^Ga-DOTA-ST8950 vs 0.29 (0.12-0.67) nM for ST8950), and similar to (by a factor of 1.9) the agonistic potency for SST5 (EC_50_ = 16 (6.7–36) vs 8.5 (3.7–19) nM, respectively). Regarding the de-iodinated analog ^nat^Ga-DOTA-ST8951, it loses massively its agonistic potency compared with ^nat^Ga-DOTA-ST8950 and the reference compounds, similar to the observation at the binding affinity studies.

**Table 3:** Agonistic potency toward SST2 and SST5 of all four ^nat^Ga-DOTA-conjugates compared to reference somatostatin analogs

	SST2	SST5
Compounds	EC_**50**_, nM (95% CI)	EC_**50**_, nM (95% CI)
Somatostatin-14^* (1)^	0.23 (0.09–0.62)	1.9 (0.77–4.5)
^nat^Ga-DOTA-ST8950	0.46 (0.23–0.92)	16 (6.7–36)
^nat^Ga-DOTA-ST8951	9.8 (3.7–26)	128 (41–512)
^nat^Ga-DOTA-TATE	0.47 (0.15–1.5)	39 (15–101)
^nat^Ga-DOTA-NOC	0.59 (0.18–1.9)	3.3 (1.3–8.5)
ST8950 ^(1)^	0.29 (0.12–0.67)	8.5 (3.7–19)
Octreotide^** (1)^	0.21 (0.12–0.36)	27 (8.1–88)
Pasireotide^** (1)^	1.1 (0.48–2.5)	0.60 (0.21–1.7)

Experiments were performed in 3 to 4 separate sessions in duplicate

*Somatostatin-14 is the natural ligand and was used as control

**Octreotide and pasireotide were used for comparison, additionally to ^nat^Ga-DOTA-TATE and ^nat^Ga-DOTA-NOC

^(1)^From [25]

### In vitro characterization

**Fig. 2:**
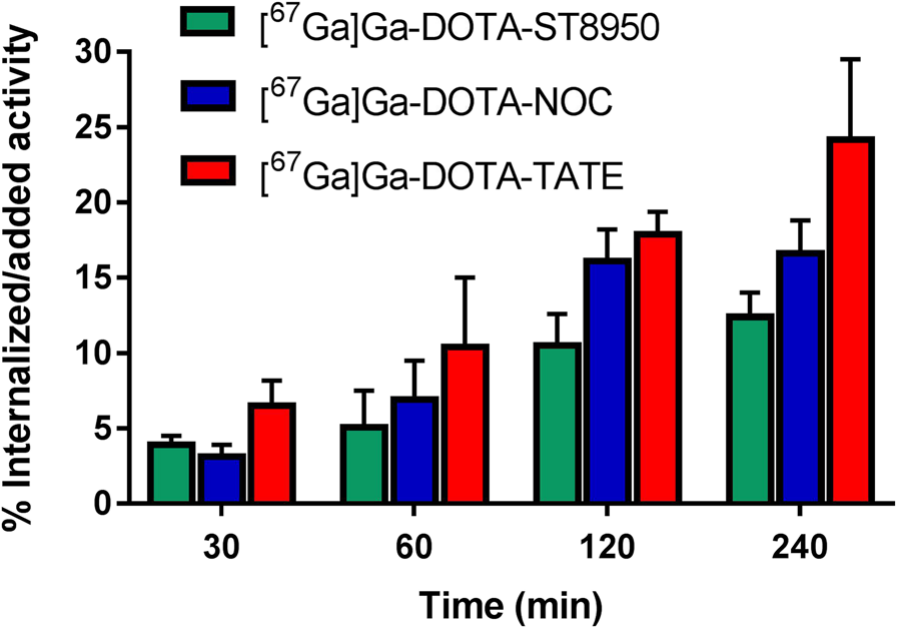
Internalization of the ^67^Ga-labeled conjugates in HEK-SST2 cells over time, expressed as % (mean ± SD) of applied activity in the cells and normalized per million cells. All values refer to specific internalization after subtracting the non-specific (measured in the presence of 1000-fold excess of SS-14) from the total internalized fraction, at each time point

[^67^Ga]Ga-DOTA-ST8950, [^67^Ga]Ga-DOTA-TATE, and [^67^Ga]Ga-DOTA-NOC showed specific and time-dependent cellular uptake on HEK-SST2 cells. The results are reported in Fig. 2. [^67^Ga]Ga-DOTA-ST8950 showed a similar (statistically not significantly different) internalization as [^67^Ga]Ga-DOTA-NOC (12.4 ± 1.6% vs 16.6 ± 2.2%, *p* = 0.0586, at 4 h), but statistically significantly lower than [^67^Ga]Ga-DOTA-TATE (12.4 ± 1.6% vs 24.2 ± 5.3%, *p* = 0.0216). The percentage of the surface-bound fraction was very low (~ 1%) in all cases, demonstrating that all surface-bound fraction is rapidly internalized inside the cells. [^67^Ga]Ga-DOTA-ST8951 had essentially no internalization on HEK-SST2, compared with the other radio-conjugates (1.1 ± 0.1%, at 4 h). None of the radio-conjugates had substantial internalization on HEK-SST5 cells ([^67^Ga]Ga-DOTA-ST8950: 1.2 ± 0.2%, [^67^Ga]Ga-DOTA-NOC 0.5 ± 0.1%, and [^67^Ga]Ga-DOTA-ST8951 < 0.5%, at 4 h). The internalization of [^67^Ga]Ga-DOTA-TATE was not evaluated on HEK-SST5, and it has no affinity to SST5.

**Fig. 3:**
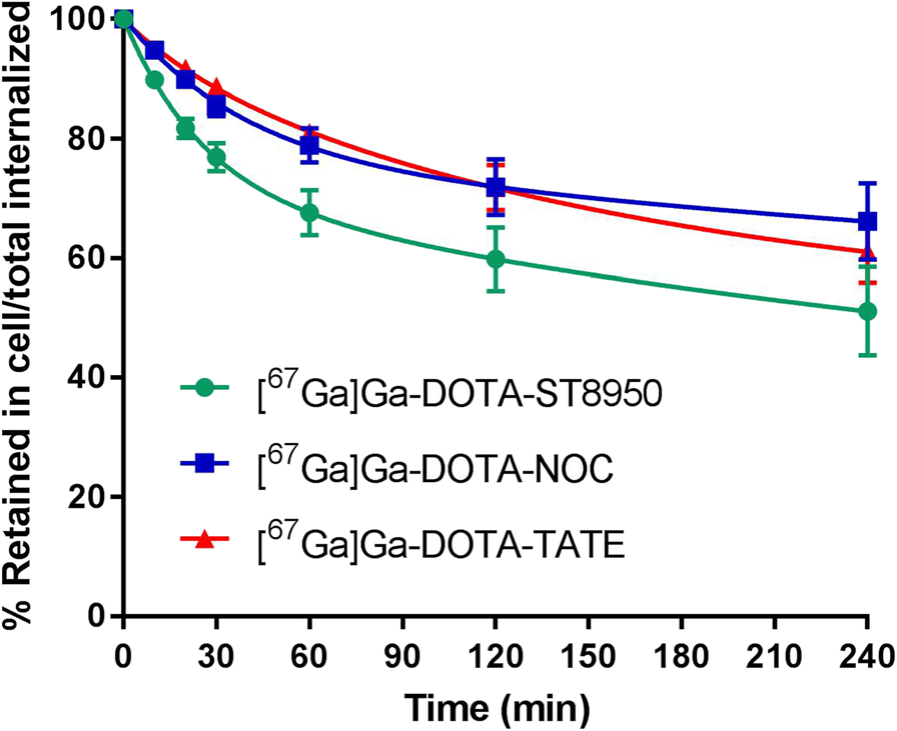
Cellular retention of the ^67^Ga-labeled conjugates in HEK-SST2 cells over time, expressed as % (mean ± SD) of the internalized activity

The results of the cellular retention of [^67^Ga]Ga-DOTA-ST8950, [^67^Ga]Ga-DOTA-TATE, and [^67^Ga]Ga-DOTA-NOC in HEK-SST2 are presented in Fig. 3. The efflux was in the same range for the three ^67^Ga-labeled conjugates. [^67^Ga]Ga-DOTA-NOC showed the lower efflux (34% after 4 h at 37 °C), while [^67^Ga]Ga-DOTA-ST8950 the highest (49% after 4 h at 37 °C); however, there was no statistically significant difference between [^67^Ga]Ga-DOTA-ST8950 and [^67^Ga]Ga-DOTA-NOC (*p* = 0.0574) or [^67^Ga]Ga-DOTA-TATE (*p* = 0.1308).

### Biodistribution studies of [^68^Ga]Ga-DOTA-ST8950 and [^68^Ga]Ga-DOTA-NOC

The biodistribution results are presented in Table 4. [^68^Ga]Ga-DOTA-ST8950 showed high accumulation in both SST2- and SST5-expressing tumors and in SST-positive tissues, such as the pancreas, stomach, and pituitary. In general, [^68^Ga]Ga-DOTA-ST8950 showed relatively long circulation in the blood, as indicated by the blood values at 1 h and 2 h p.i. (1.9 ± 0.6 and 0.8 ± 0.2 %IA/g, respectively). The accumulation in the kidneys (14 ± 4 %IA/g at 1 h p.i., remaining at the same level after 2 h) indicates urinary excretion and renal retention. The liver uptake of [^68^Ga]Ga-DOTA-ST8950 is rather high (6.4 ± 1.9 %IA/g), compared to [^68^Ga]Ga-DOTA-NOC (2.3 ± 0.7 %IA/g). The significantly low tumor uptake of [^68^Ga]Ga-DOTA-ST8950 in HEK-SST-negative xenografts (1.4 ± 0.5 %IA/g), versus 26 ± 8 %IA/g in HEK-SST2 tumors and 15 ± 6 %IA/g in HEK-SST5 tumors at 1 h p.i. confirms the receptor-mediated uptake (specificity) of [^68^Ga]Ga-DOTA-ST8950.

**Table 4:** Biodistribution results of [^68^Ga[Ga-DOTA-ST8950 at 1 and 2 h p.i. and [^68^Ga]Ga-DOTA-NOC at 1 h p.i

[^**68**^Ga]Ga-DOTA-ST8950				[^**68**^Ga]Ga-DOTA-NOC
Organ	1 h^**#**^	2 h^**&**^	1 h (negative)^**‡**^	1 h^**&**^
Blood	1.9 ± 0.6	0.8 ± 0.2	1.5 ± 0.2	1.1 ± 0.4
Heart	1.3 ± 0.4	0.8 ± 0.2	1.1 ± 0.2	0.7 ± 0.2
Lung	5.4 ± 1.6	3.5 ± 0.7	4.6 ± 0.8	3.3 ± 1.4
Liver	6.4 ± 1.9	7.3 ± 1.5	6.2 ± 0.6	2.3 ± 0.7
Pancreas	8.5 ± 4.5	8.6 ± 2.1	7.4 ± 1.9	16 ± 3
Spleen	1.9 ± 0.6	1.5 ± 0.3	1.6 ± 0.1	0.8 ± 0.2
Stomach	7.5 ± 3.0	8.4 ± 1.2	6.0 ± 1.6	12 ± 2
Intestine	2.4 ± 1.3	2.7 ± 0.3	2.5 ± 0.2	3.3 ± 0.7
Adrenal	4.5 ± 0.9	4.3 ± 1.0	3.4 ± 0.7	4.6 ± 1.0
Kidney	14 ± 4	14 ± 2	12 ± 1	9.8 ± 3.2
Muscle	0.9 ± 0.3	0.6 ± 0.1	0.5 ± 0.1	0.5 ± 0.2
Femur	2.0 ± 0.6	2.4 ± 0.8	1.1 ± 0.1	1.7 ± 0.8
Pituitary	8.5 ± 1.9	7.8 ± 1.2	n.d.	8.2 ± 2.8
**SST2 tumor**	26 ± 8	32 ± 7	–	30 ± 8
**SST5 tumor**	15 ± 6	14 ± 3	–	12 ± 5
SST(-) tumor	–	–	1.4 ± 0.5	–

Results are expressed as the mean of the % injected activity per gram of tissue (%IA/g) ± standard deviation (SD). The peptide mass and activity injected were 100 pmol and 2.5 MBq, for both radio-conjugates

*SST(-)* somatostatin receptor negative

^#^*n* = 8, ^**&**^*n* = 5, ^‡^*n* = 3, n.d. not determined

The biodistribution of [^68^Ga]Ga-DOTA-NOC was similar to [^68^Ga]Ga-DOTA-ST8950 at 1 h p.i., with high and specific accumulation in SST2 and SST5 tumors and in SST-positive tissues. However, [^68^Ga]Ga-DOTA-ST8950 showed slightly higher blood values and higher kidney and liver uptake, compared to [^68^Ga]Ga-DOTA-NOC, which had in turn higher accumulation in the SST-positive organs, such as the pancreas and stomach.

### PET/CT imaging of [^68^Ga]Ga-DOTA-ST8950 and [^68^Ga]Ga-DOTA-NOC

**Fig. 4:**
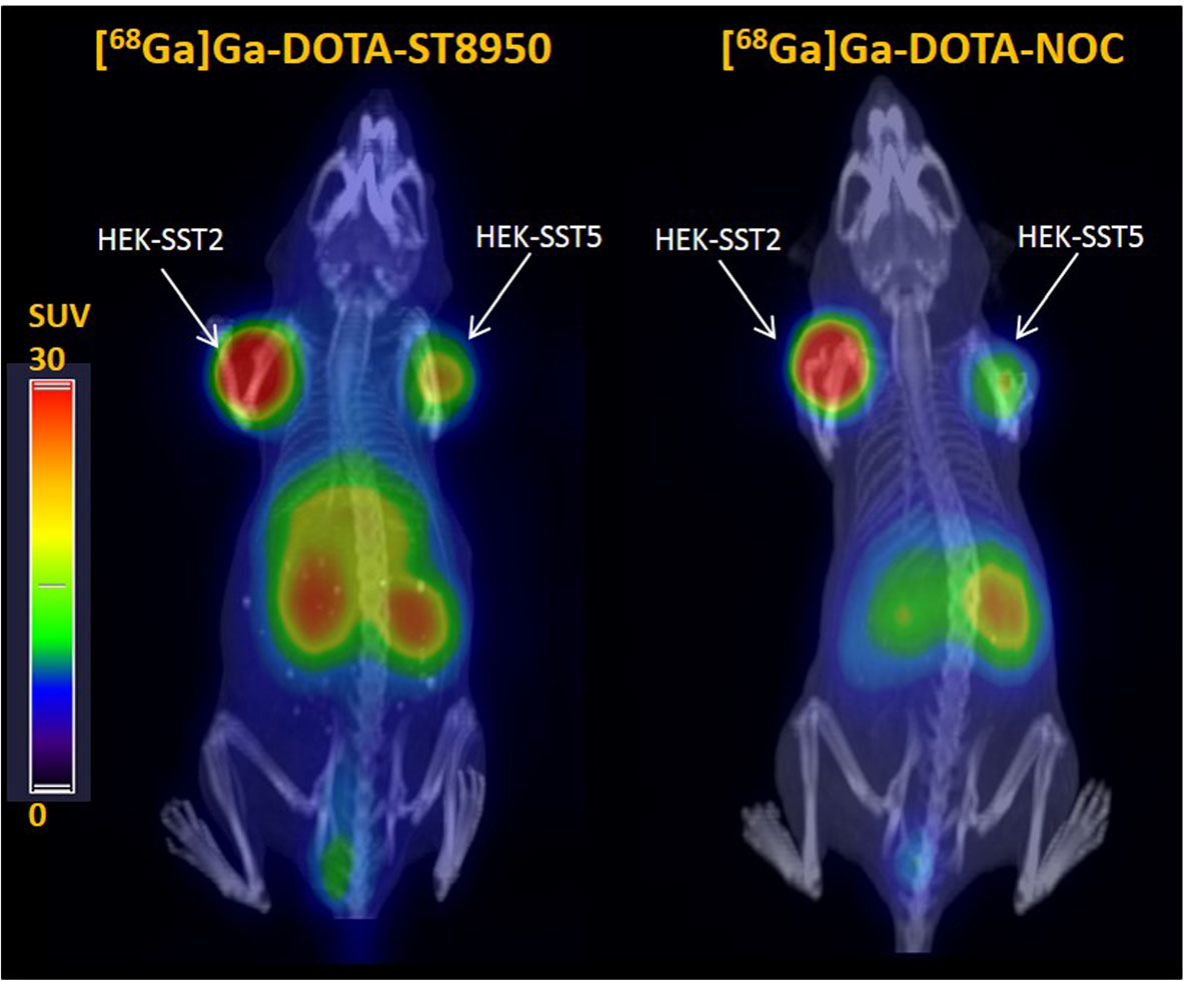
Maximum intensity projection (MIP) PET/CT images of [^68^Ga]Ga-DOTA-ST8950 and [^68^Ga]Ga-DOTA-NOC (100 μL/100 pmol/5 MBq, apparent molar activity of 50 MBq/nmol) in a dual SST2- and SST5-expressing tumor mouse model, 1 h after the injection of the radiotracer (SUV: standard uptake value)

PET/CT images of [^68^Ga]Ga-DOTA-ST8950 and [^68^Ga]Ga-DOTA-NOC at 1 h p.i. revealed high image contrast with clear visualization of both SST2 and SST5 tumors (Fig. 4). The highest tracer uptake is visible in the tumors and in the kidneys. Accumulation of [^68^Ga]Ga-DOTA-ST8950 is also detected in the liver, in agreement with the biodistribution data, though lower than in the kidneys and the tumors.

## Discussion

Various expression and co-expression patterns have been described for the 5 somatostatin receptor subtypes (SST1-5), depending on the tumor type and origin [5, 6, 28]. Interestingly, tumor area lacking expression of a given subtype may be populated by another one [4, 5, 8]. Hence, somatostatin analogs with affinity to more than one receptor subtypes are of great interest as they address receptor subtype co-expression and heterogeneous expression patterns.

Two independent studies comparing the diagnostic efficacy [^68^Ga]Ga-DOTA-NOC, a somatostatin analog with a high affinity for SST2 and SST5 and a lower affinity for SST3, with the SST2-selective [^68^Ga]Ga-DOTA-TATE in NET patients, provided controversial results on the clinical outcome of multi-receptor subtype targeting. Kabasakal et al. [29], concluded that even though the images have comparable diagnostic accuracy, [^68^Ga]Ga-DOTA-TATE detected more lesions. Contrary to this, Wild et al. [30] reported that [^68^Ga]Ga-DOTA-NOC detected significantly more lesions than [^68^Ga]Ga-DOTA-TATE (sensitivity: 93.5 vs 85.5%) and it changed the clinical management in 17% of the studied patients. Recently, Lamarca et al. [31] confirmed the role of [^68^Ga]Ga-DOTA-NOC PET imaging for the optimization of the clinical management in lung carcinoid patients. Overall, the clinical data support that multi-receptor subtype targeting is relevant for improving the diagnostic accuracy and sensitivity of PET imaging of SST-expressing tumors. Therefore, effort needs to be made for developing new radiotracers in this direction.

With the aim to develop ^68^Ga-tracers for combined SST2 and SST5 targeting, we focused on the series of compounds reported by Moore et al. [24], who used synthetic amino acids, among them iodo-substituted ones, to improve binding affinities. There are several cases in the literature where (radio)iodination of somatostatin analogs either did not affect or improve the binding affinity and/or potency [8, 32, 33]. In the series of Moore et al., iodination at position 3 (3-iodo-Tyr^3^, peptide #6 in reference [24]) showed improved affinity to SST5 by an order of magnitude, followed unluckily, by a 4-fold reduction in the affinity to SST2. Similar observations on SST5 were reported by Schotellius et al. [33]. Iodination at position 3 of DOTA-TATE (DOTA-3-iodo-Tyr^3^-octreotate: HA-TATE) enhanced the affinity of ^nat^Ga-DOTA-HA-TATE to SST5, compared with ^nat^Ga-DOTA-TATE (IC_50_ = 102 ± 65 vs > 1000 nM, respectively), but did not affect the affinity to SST2, contrary to Moore et al. Taken together, the two studies indicate that iodo-substitution of Tyr^3^ on the octreotide motif improves affinity to SST5.

Modification of ST8950 at the N-terminal by coupling of DOTA and complexation with Ga^3+^ does not affect the affinity for SST2, while reduces the affinity for SST5 by a factor of approx. 2. Nevertheless, ^nat^Ga-DOTA-ST8950 retains its affinity in sub- (SST2) or one-digit (SST5) nanomolar range. It worth mentioning that the determined IC_50_ of ST8950 is in very good agreement with the results reported by Moore et al. [24] (0.6 for SST2 and 0.7 nM for SST5). Conversely, the de-iodinated analog ST8951 (IC_50_ = 1.6 nM for SST2 and 14 nM for SST5 in reference [24]) after the coupling of the chelate ^nat^Ga-DOTA losses massively its affinity for both, SST2 and SST5 (IC_50_ = 7.5 and 24 nM, respectively). The effect is more prominent in the SST2, for which ST8951 shows higher selectively. In addition, we evaluated to which extend ST8950 and ST8951 retain their agonistic potencies after Ga-DOTA conjugation, in light of examples in the literature indicating that modifications like DOTA conjugation can change the function of a somatostatin analog from an antagonist to an agonist [34]. The agonistic potencies of ^nat^Ga-DOTA-ST8950 and ^nat^Ga-DOTA-ST8951 followed an identical trend as their affinities to both SST2 and for SST5.

[^67^Ga]Ga-DOTA-ST8950 had similar internalization rate as [^67^Ga]Ga-DOTA-NOC, but significantly lower than [^67^Ga]Ga-DOTA-TATE in HEK-SST2 cells. All three radiotracers had very low surface-bound fraction, confirming their agonistic nature that leads to instant internalization of the radiotracer upon binding to the receptor on the cell surface. Four hours after internalization, half of the [^67^Ga]Ga-DOTA-ST8950 still remains inside the cells. There was no statistically significant difference in the efflux rate between [^67^Ga]Ga-DOTA-ST8950 and [^67^Ga]Ga-DOTA-NOC or [^67^Ga]Ga-DOTA-TATE. [^67^Ga]Ga-DOTA-ST8951 showed essentially no internalization in HEK-SST2. The inability of [^67^Ga]Ga-DOTA-ST8951 to bind and internalize on SST2-expressing cells, together with its loss of affinity for SST2 and SST5, led us to exclude [^67/68^Ga]Ga-DOTA-ST8951 from further studies.

In vitro experiments on HEK-SST5 did not show any internalization (neither cell surface-bound) for any of the tested radiotracers. This may be due to the particular cellular distribution and trafficking of SST5 [35, 36] and not due to the radiotracers. This phenomenon was also observed by others using somatostatin analogs with a high affinity for SST5 [10, 35]. Cescato et al. [35] showed that SST5 internalization can be induced by natural somatostatin peptides but not by synthetic high-affinity SST5 agonists. Indeed, Maina et al showed in vitro internalization on HEK-SST5 of an ^111^In-labeled modified analog of the natural SS-28 [22], even though very low (approx. 2.5% after 1 h at 37 °C) and unusually high (50%) nonspecific portion. Our data are in line with the published findings. Importantly, the lack of in vitro internalization in SST5-expressing cells does not exclude the accumulation of the radiotracer in SST5-expressing tumors in vivo. Our in vivo data prove this.

The in vivo distribution of [^68^Ga]Ga-DOTA-ST8950 is representative of radiolabeled somatostatin analogs, regarding the accumulation in SST-positive tissues, such as the stomach, the pancreas, and the pituitary. [^68^Ga]Ga-DOTA-ST8950 showed high uptake in both SST2- and SST5-expressing tumors, similar to [^68^Ga]Ga-DOTA-NOC (*p* = 0.4630 for SST2 and *p* = 0.3282 for SST5), proven to be receptor subtype mediated by the 90% reduction found on the SST-negative tumor. The kidneys were the second tissue after the tumors accumulating radioactivity, which was expected due to the renal excretion of this class of radiotracers. Unluckily, the lipophilic character of [^68^Ga]Ga-DOTA-ST8950 was reflected on its biodistribution profile, with rather high blood and liver values. Nevertheless, when we compared with the ^68^Ga-labeled pasireotide which also targets SST2 and SST5 (referred as ^68^Ga-DOTA-SOM230 in [15, 16]) in the same animal model, [^68^Ga]Ga-DOTA-ST8950 has advantages in terms of lower blood values (1.9 ± 0.6 vs 4.1 ± 0.9 %IA/g at 1 h p.i.) and liver uptake (6.4 ± 1.9 vs 12.9 ± 2.2 %IA/g at 1 h p.i.). Liu et al. confirmed similarly high blood values and even higher liver uptake for the ^68^Ga-labeled pasireotide (referred as ^68^Ga-DOTA-PA1 in [17]). However, [^68^Ga]Ga-DOTA-ST8950 has certain limitations when compared with the clinically used [^68^Ga]Ga-DOTA-NOC and [^68^Ga]Ga-DOTA-TATE. The blood and liver values of [^68^Ga]Ga-DOTA-ST8950 are higher than [^68^Ga]Ga-DOTA-NOC (Table 4, *p* = 0.0141 and *p* = 0.0005, respectively) and much higher when compared with our previous data on [^68^Ga]Ga-DOTA-TATE [37] (blood: 1.9 ± 0.6 vs 0.4 ± 0.0 %IA/g and liver 6.4 ± 1.9 vs 0.4 ± 0.2 %IA/g, respectively). On the other hand, [^68^Ga]Ga-DOTA-NOC demonstrated higher uptake in the SST-positive stomach and pancreas, while [^68^Ga]Ga-DOTA-TATE cannot be used for imaging SST5-expressing tumors as it is unable to bind to this receptor subtype.

PET/CT imaging is reflecting the biodistribution data, with clear visualization of SST2- and SST5-expressing tumors and high image contrast for [^68^Ga]Ga-DOTA-ST8950 and for [^68^Ga]Ga-DOTA-NOC. The higher kidney uptake of [^68^Ga]Ga-DOTA-ST8950, compared with [^68^Ga]Ga-DOTA-NOC (*p* = 0.0399), is of no concern for a diagnostic tracer. However, the accumulation of [^68^Ga]Ga-DOTA-ST8950 in the liver is a drawback. Especially when recognizing that the liver is the first site of metastasis of NETs, and therefore, low background activity is needed for a good image contrast and diagnostic accuracy. Two approaches are considered to circumvent this problem: (a) a chemical approach that involves modification of the structure by introducing hydrophilic spacers and/or amino acids and (b) a pharmacological approach by enhancing the tumor uptake via epigenetic receptor upregulation [38], improving tumor-to-liver ratio. The second approach is mainly considered for the therapeutic counterpart [^177^Lu]Lu-DOTA-ST8950.

## Conclusion

The preclinical evaluation of the 2-iodo-substituted somatostatin analog [^68^Ga]Ga-DOTA-ST8950 reveals its potential as PET tracer for in vivo imaging of SST2- and SST5-expressing tumors, which may be of interest for gastroenteropancreatic neuroendocrine tumors, pituitary tumors, and gastric cancers. Its in vivo uptake in the tumors compares favorably with the uptake of the clinically used [^68^Ga]Ga-DOTA-NOC, but its high accumulation in the liver remains a challenge for clinical translation. While iodination in positions 1 and 3 seemed not to be a prerequisite for a good binding affinity of ST8950 and of the de-iodinated ST8951 to SST2 and SST5, this is proven to be essential in their [^68^Ga]Ga-DOTA-chelated versions. [^68^Ga]Ga-DOTA-ST8951 lost its affinity and potency for both subtypes, and it is disqualified for usage as a PET tracer.

## Data Availability

The datasets used and analyzed during the current study are available from the corresponding author on reasonable request.

## Abbreviations

CT: Computed tomography
DMEM: Dulbecco’s modified Eagle’s medium
DOTA: 1,4,7,10-Tetraazacyclododecane-1,4,7,10-tetraacetic acid
DTPA: Diethylenetriaminepentaacetic acid
ESI-MS: Electrospray ionization mass spectroscopy
FBS: Fetal bovine serum
NET: Neuroendocrine tumor
PBS: Phosphate-buffered saline
PET: Positron emission tomography
p.i.: Post-injection
RP-HPLC: Reverse phase high-performance liquid chromatography
SST: Somatostatin receptor
SS-14: Somatostatin-14
SS-28: Somatostatin-28
